# Cancer research using organoid technology

**DOI:** 10.1007/s00109-020-01990-z

**Published:** 2020-10-14

**Authors:** Kai Kretzschmar

**Affiliations:** Mildred Scheel Early Career Centre (MSNZ) for Cancer Research Würzburg, University Hospital Würzburg, MSNZ/IZKF, Josef-Schneider-Str. 2, 97080 Würzburg, Germany

**Keywords:** Cancer, Organoids, 3D culture, Pre-clinical models, Personalised medicine, Drug screening, Immuno-oncology

## Abstract

Organoid technology has rapidly transformed basic biomedical research and contributed to significant discoveries in the last decade. With the application of protocols to generate organoids from cancer tissue, organoid technology has opened up new opportunities for cancer research and therapy. Using organoid cultures derived from healthy tissues, different aspects of tumour initiation and progression are widely studied including the role of pathogens or specific cancer genes. Cancer organoid cultures, on the other hand, are applied to generate biobanks, perform drug screens, and study mutational signatures. With the incorporation of cellular components of the tumour microenvironment such as immune cells into the organoid cultures, the technology is now also exploited in the rapidly advancing field of immuno-oncology. In this review, I discuss how organoid technology is currently being utilised in cancer research and what obstacles are still to be overcome for its broader use in anti-cancer therapy.

## Introduction

Cancer remains a major threat to quality of life with significant risks of morbidity and mortality worldwide [1], despite extraordinary progress made in cancer research, prevention, detection, and therapy in the past decades. For example in the USA, incidences of the prominent cancer types of lung or colorectal cancer are decreasing partially due to increased knowledge on cancer biology and, hence, improved prevention, while incidence rates of other cancer types such as liver or oral cancers are increasing [2]. However, cancer is a heterogenous disease with a broad range of types and subtypes, which can be defined based on their anatomical location, histological appearance, and genetical makeup. In order to guide the way to improved targeted therapy, pre-clinical model systems are essential to better capture the inter- and intra-tumour heterogeneity. For instance, animal cancer models, in particular genetically engineered mouse models (GEMMs), have provided significant insights into the cellular and genetic basis of cancer [3]. However, their application is rather costly, time-consuming, and often cannot be translated into therapy, due to major differences to human pathology and tumourigenesis (reviewed in [4, 5]). Using human cancer models such as cancer cell lines and patient-derived xenografts (PDTXs), some of these limitations were overcome: in principle, these models can be generated from a larger cohort of patients better presenting the inter-tumour heterogeneity [6]. Yet, these models also have significant drawbacks. Cancer cell lines often do not sufficiently retain the intra-tumour cellular and genetic heterogeneity in vitro, as only robust and colony-forming cells (i.e. clones) can be maintained in culture long-term, which are frequently genetically instable [6, 7]. Furthermore, cancer cell lines are devoid of the cellular microenvironment of the tumour in vivo, including the tumour stroma as well as immune infiltrate [6]. In most cases, cancer cell lines also lack matched cell lines established from normal tissue as reference control [6]. PDTXs are generated by transplanting primary patient tumour material into immunocompromised mice [8]. As such, PDTX models allow for the spontaneous development of a tumour stroma of murine origin and the investigation of metastasis formation [6, 8]. PDTX-based approaches therefore do model some critical aspects of the tumour and its microenvironment. Yet, PDTXs still lack the human-specific immune components, require the use of animals (potentially causing mouse-specific features not found in human cancer), and are both expensive and time-consuming (reviewed in [9, 10]).

**Fig. 1:**
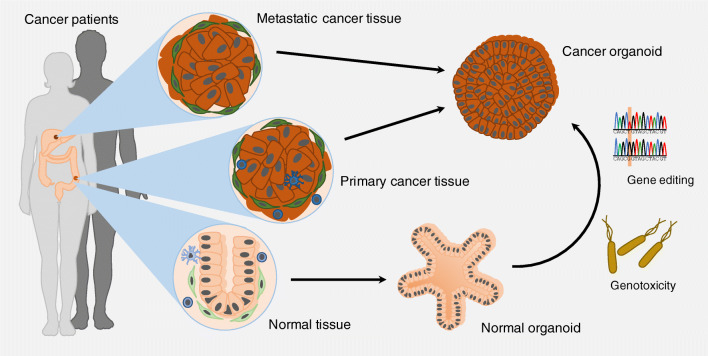
Generation of patient-derived normal and cancer organoids. Patient-derived cancer organoids can be established from primary and metastatic cancer tissue. Matched normal organoids can be generated from normal tissue. Through gene editing, normal organoids may be transformed into cancer organoids. By exposure to genotoxic factors, it may also allow for malignant transformation of normal organoids in vitro, as a recent study showed that normal organoids incubated with genotoxic bacteria acquired mutational signature characteristic of cancer subsets [63]. Some images were modified from the medical art database at https://smart.servier.com/

A promising alternative to these conventional cancer models is based on the discovery that adult stem cells (ASCs) proliferate and spontaneously self-organise into three-dimensional (3D) organotypic cellular structures—so-called organoids—in culture, when they are embedded into a hydrogel rich in extracellular matrix (ECM) proteins such as Matrigel or Basement Membrane Extract (BME) [11]. A key to organoid technology is the tissue-specific growth factor cocktail provided to the culture. In the case of the first organoid model system—murine small intestinal organoids generated from *Lgr5*^+^ intestinal epithelial stem cells—the growth medium contained the Wnt pathway agonist and ligand of LGR5 R-spondin-1, epidermal growth factor, and the bone morphogenetic protein (BMP) pathway inhibitor Noggin [11]. By designing a growth factor cocktail specific to each stem cell type, tissue, and species, the protocol was adapted to allow for the generation of organoid cultures from other murine and human epithelial tissues (Fig. 1; reviewed in [12, 13]) such as the bladder [14], breast [15], colon (and rectum) [16, 17], endometrium [18], fallopian tubes [19], kidney [20], liver [21, 22], lung [23], oesophagus [24], oral mucosa [25], pancreas [26], prostate [27, 28], salivary gland [29], skin epidermis [30], stomach [31], and taste buds [32], and, most recently, even non-mammalian tissue such as snake venom glands [33]. As such, it has also been possible to generate ASC-enriched epithelial organoids from tissue pieces [11] or tissue pieces containing both epithelial and stromal cells [34]. Organoids allow for the long-term expansion of stem cells and their spontaneous differentiation into specialised tissue cells, most prominently seen in the crypt–villus differentiation observed in murine small intestinal organoids [11, 35] or stratification and cornification described for murine skin epidermal organoids [30] as well as murine and human oral epithelial organoids [25]. Long-term analyses further suggest that ASC-derived organoids remain largely phenotypically and genetically stable, reflecting their tissue of origin [36]. Comparative mutational analysis of organoid cultures generated from different murine and human tissues further demonstrated that tissue-specific mutational signatures can be defined using organoids [36–38]. In addition, organoid cultures are amenable to a wide range of experimental tools, including single-cell transcriptomics [39], gene editing and tagging [40], (whole-mount) imaging [41], xenotransplantation [42], and co-culture with other cells such as immune cells (reviewed in [43]). Another complementary approach is to generate organoids using pluripotent stem cells (PSCs), namely embryonic stem cells or induced pluripotent stem cells (iPSCs) (reviewed in [12, 13]). PSC-derived organoids reflecting various types of tissues and organs including the brain [44], intestine [45], kidney [46, 47], and retina [48, 49] have been described. As PSC-derived organoids typically remain phenotypically and transcriptionally immature (reviewed in [50]) and, therefore, are more similar to embryonic-like tissue, their use for cancer research has been limited so far (reviewed in [51]). Another important disadvantage of PSC-derived organoids in cancer research is the lack of acquired cancer gene mutations in individual tumour organoid lines. These models are therefore less attractive for biobanking or drug screening, in contrast to organoids directly derived from tumour biopsies that preserve cancer gene mutations in culture. However, improved methods may allow for broader utilisation of PSC-derived organoid models in cancer research in the future.

In this review, I discuss the use of human ASC-based organoid technology in basic and translational cancer research. I explore how epithelial organoid cultures serve as a base to study the processes of tumourigenesis and metastasis, including the role of the tissue microenvironment. I further highlight the use of cancer organoids generated from primary patient material in biobanks for drug discovery, personalised anti-cancer therapy, and immuno-oncology. I close by discussing challenges of organoid technology to be overcome to allow its wider use in the clinic and future prospects of its further exploitation in cancer research.

## Genotoxic factors and cancer initiation

Cancer develops in a multistep process from normal tissue by acquisitions of somatic mutations in so-called cancer driver genes [52]. Cells throughout our body are continuously challenged by different endogenous or exogenous genotoxic factors that can damage their DNA [53, 54]. While most of the DNA damage induced is being continuously repaired by one of the DNA repair pathways, errors in the DNA repair process may result in the gain of mutations at a low frequency [52, 55]. However, prolonged exposure to such genotoxic factors may further increase the risk of developing cancer. The International Agency for Research on Cancer (IARC) of the World Health Organization (WHO) has published a list of exogenous cancer hazards, including physical agents such as ionising radiation (i.e. gamma rays, X-rays, and higher spectrum ultraviolet light), carcinogenic chemicals, and certain types of pathogenic infections (‘IARC monographs’, https://monographs.iarc.fr/). While animal models are widely used for research into agents associated with an increased cancer risk, it is often rather difficult to define a mechanism of action. In many cases, it is therefore not possible to determine whether a suspected carcinogenic agent can be classified as carcinogen to humans [54]. As epithelial organoid cultures remain essentially phenotypically and genetically stable long-term, they are an excellent model system to study the genotoxic potential of different agents. Attempting this strategy, a recent study described the testing of different genotoxic chemicals, namely ethyl methanesulfonate (EMS), acrylamide (AA), and 7,12-dimethylbutylamine (DMBA), on mouse-derived epithelial organoids for their potential to generated tumours upon transplantation into nude mice [56]. Murine mammary gland organoids with partial loss of *Trp53* treated with DMBA displayed tumourigenicity upon transplantation, while wild-type *Trp53* counterparts did not develop tumours in vivo, in line with earlier work done in mouse models. Murine lung organoids, irrespective of their *Trp53* status, developed tumours in vivo following exposure to EMS and AA [56], overall demonstrating that organoids may be used as chemical carcinogenicity studies. Further improvements should aim to allow for the assessment of genotoxic potential of chemicals in human tissue–derived organoids with a setup that is entirely in vitro.

Apart from ionising radiation and carcinogenic chemicals, pathogenic infections have been linked to tumourigenesis. Again, organoids may allow for the investigation of pathogenic infections and their contribution to cancer development. Different organoid–pathogen co-culture protocols have been described (reviewed in [57, 58]). For instance, gastric organoids have been cultured with *Helicobacter pylori* [31], a known pathogen populating the stomach that has long been linked to gastric cancer [59]. Other studies demonstrated, for example, that human noroviruses can replicate in human small intestinal organoid cultures [60]; that human papilloma viruses (HPV), described as oncogenic factors in subtypes of head and neck cancer [61], can infect human oral epithelial organoids [25]; and that the parasite *Cryptosporidium* can complete its life cycle in human small intestinal and lung organoids [62]. A recent study addressed whether the microbiome directly contributes to tumourigenesis using organoid technology [63], as it has long been suggested that gut microbiota may have been involved in colorectal cancer (CRC) development [64]. The authors repeatedly injected a strain of genotoxic *Escherichia coli* (*pks E. coli*) expressing an enzyme that synthesises colibactin into the lumen of cystic human intestinal organoids for a period of up to 5 months. In line with a previous report demonstrating that colibactin induces double-strand breaks in cultured cells [65], the authors found that organoids co-cultured with *pks E. coli* had increased levels of DNA damage [63]. Subsequently, whole-genome sequencing (WGS) was performed on the DNA collected from organoids before and after exposure to *pks E. coli*. Data were then compared with those obtained from organoid lines exposed to an isogenic control strain of *E. coli* incapable of producing active colibactin (*pks*-mutant *E. coli*) [63]. Organoids exposed to *pks E. coli* acquired distinct mutational signatures, which were present neither in organoids before bacteria co-culture nor in those co-cultured with *pks*-mutant *E. coli*. Assessment of more than 5000 genomes of human cancer metastases revealed that the same mutational signatures were present in 11% of CRC genomes as well as genomes of other cancers including head and neck cancers and urinary tract cancers [63]. In a second CRC-specific patient cohort, the *pks E. coli*-induced mutational signatures were found in more than 20% of all CRC genomes analysed [63]. This suggests that pathogenic bacteria may directly contribute to malignant transformation (Fig. 1). Organoids that grow as cystic spheroids such as human intestinal organoids are well suited for studying host–pathogen interactions using intra-luminal injections [62, 63]. However, future progress may expand possibilities to study organoid–pathogen interactions in basic cancer research. For instance, modelling more complex communities of the gut microbiota in organoid–bacteria co-cultures may allow for the functional validation of the microbiome alternations linked to CRC formation and progression [66, 67].

## Cancer organoids and ‘living’ biobanks of cancer

**Fig. 2:**
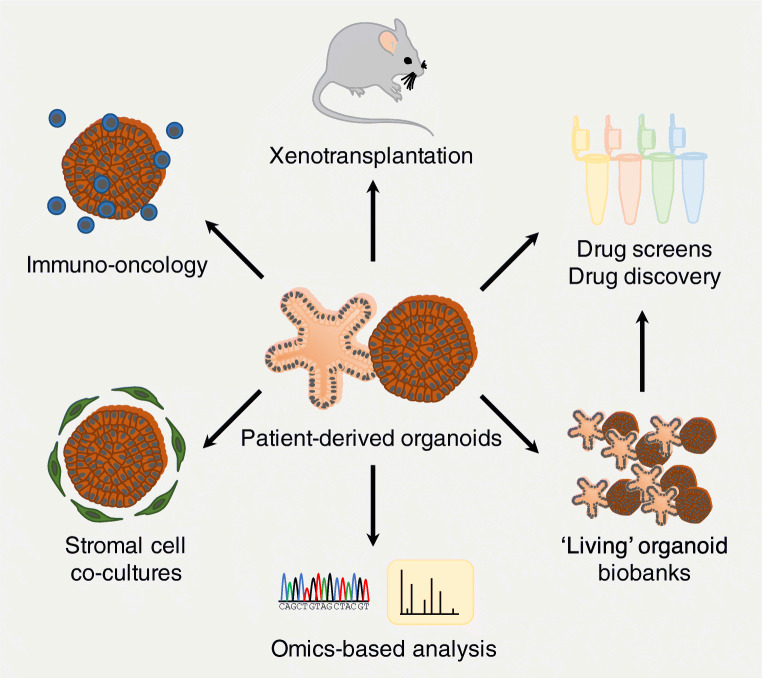
Utilisation of patient-derived organoids in cancer research. Patient-derived (cancer) organoids have already been used to generate ‘living’ organoid biobanks that can be exposed to different drugs for efficacy screenings and drug discovery validations. Organoids have further been used to study inter- and intra-tumour heterogeneity by analysis of mutational signatures, gene expression patterns, or proteomics. By transplanting cancer organoids into mice, tumour cell invasiveness and potential to metastasise can be tested. Finally, approaches to incorporate cells of the tumour microenvironment such as stromal cells (such as cancer-associated fibroblasts) or immune cells (i.e. immuno-oncology) are being developed. Some images were modified from the medical art database at https://smart.servier.com/

The adaption of protocols to generate organoids from human ASCs allowed the derivation of cancer organoids from patient material, typically surgical specimen or needle biopsies [68]. Establishment of cancer organoid cultures has been described for primary and/or metastatic tumour tissue sampled from the bladder [14, 69], brain [70, 71], breast [15], colon [17, 72–74], endometrium [18, 75], head and neck [25, 68], kidney [76, 77], liver [78], lung [23], oesophagus [17], ovaries [79, 80], pancreas [26, 81, 82], prostate [83], rectum [72, 84], and stomach [85, 86] (Fig. 1). Overgrowth or contamination by normal (epithelial) cells is a major drawback for the generation of organoid cultures containing only epithelial tumour cells [68, 87]. For instance, based on the observation that cultures lacked copy number alterations and were free of any mutations in fifty common cancer-associated genes, Gao et al. reported that several of their organoid cultures generated from prostate cancer metastases into lung and liver had likely been overgrown by normal epithelial cells [83]. This observation highlights two considerations when establishing cancer organoid cultures. Firstly, due to higher rates of cell death by mitotic catastrophes and other aberrations [40, 88], cancer organoids often grow slower than normal organoids, and, secondly, many normal organoid cultures thrive under surprisingly simple growth conditions or require similar conditions as their cancerous counterparts. To avoid this, different strategies have been developed. On the one hand, cancer organoid cultures may be established from metastatic tissue, ideally, taken from sites devoid of normal epithelial cells such as lymph node biopsies, bone biopsies, or ascites fluid. On the other hand, pure cancer organoid cultures may be generated by providing a minimal or selective medium inhibiting the growth of normal epithelial cells. Activating mutations in the Wnt/β-catenin signalling pathway are found in about 95% of all cases of CRC. Therefore, pure CRC organoid cultures have been achieved by removal of Wnt pathway stimulants such as Wnt ligands and R-spondins from the growth medium [17, 72, 88]. Withdrawal of other growth factors may be used to select for tumour cells when mutations in other specific signalling pathway have been identified. For instance, epidermal growth factor (EGF) withdrawal allows for the selection of rat sarcoma viral oncogene homologue (RAS) mutants, hence EGF receptor (EGFR)–signalling independent tumour cells [88]. In accordance, combinatory withdrawal of growth medium components has been used to select for CRC subtypes with specific growth factor independencies [73, 88]. Furthermore, cancer organoid cultures have been robustly purified from non-small cell lung carcinomas, which are frequently mutant for *TP53* by selection with the MDM2 agonist Nutlin-3 [23]. In line with observations reported for organoids established from normal (i.e. healthy) primary material, cancer organoids histologically, transcriptionally, and genetically largely retain the bulk characteristics of the tumour epithelium of origin. Based on cancer organoid protocols, large efforts have been made to generate ‘living’ biobanks of patient-derived cancer organoids (Fig. 2; Table 1 and references therein), often with their matched normal counterparts. An important consideration here is the use of patient-matched pairs of normal and cancer organoid lines for subsequent analyses such as drug screenings (see “Personalised anti-cancer therapy”). Such matched pairing of organoid lines may allow accounting for genetic and phenotypic variation among normal human epithelial organoids derived from different patients [36–38, 89], which may even exceed the effects of single cancer gene mutations. As the complexity of cancer results in cancer types being further broken down into subtypes, it is important to note that the majority of these biobanks provide cancer organoid cultures representing different cancer subtypes. Overall, more and more cancer biobanks are being described. However, as most of the existing cancer organoid protocols were developed for epithelial carcinomas, future research should aim at generating more organoid cultures from non-epithelial cancers as those recently described for glioblastoma [70] and rhabdoid tumours of the kidney [76].

**Table 1:** Overview of published patient-derived cancer organoid biobanks. Listed are only studies describing cancer organoid collections with cancer organoid lines established from more than three patients

Cancer type	Source				Validation and analysis						Therapy testing				Year of publication	References
	Primary cancer tissue	Metastatic cancer tissue	Other tumour tissue	Matched normal tissue	Tumour-specific medium	Histology	DNA^a^	RNA^b^	Xenografting	Other analysis^c^	Drug screen	Radiotherapy	Immunotherapy	Clinical response		
Biliary tract cancer	●			●		●	E	M/P	●		●				2019	[139]
Bladder cancer	●					●	T/E	R	●		●				2018	[69]
	●			●		●		R			●				2019	[14]
Breast cancer	●	●				●	G	R	●		●				2018	[15]
Colorectal cancer	●			●	●	●	E	M	●	Pro	●				2015	[72, 124, 125]
		●					E								2015	[74]
	●	●		●	●	●	T/E	R	●						2016	[73]
	●	●				●					●				2016	[126]
	●	●				●	E/G	R			●				2017	[127]
		●					G				●			●	2019	[104]
Endometrial cancer	●	●	●	●		●	E	R	●	EM	●				2019	[75]
Gastric cancer	●	●				●	G				●				2018	[128]
	●	●	●	●	●	●	E	R			●			●	2018	[85]
	●					●	G	R	●		●				2019	[129]
Gastrointestinal cancer^d^		●				●	G	R	●		●			●	2018	[87]
Glioblastoma	●					●	E	R/scR	●		●	●	●		2020	[70]
Head and neck cancer	●			●		●	T/E	R	●	EM	●	●		●	2019	[25]
Kidney cancer	●			●		●	E	R	●		●				2019	[77]
	●	●		●		●	G	scR		Me	●				2020	[76]
Liver cancer	●				●	●	E	R	●		●				2017	[78]
	●					●	E				●				2019	[130]
Lung cancer	●			●	●	●	E		●			●			2019	[131]
	●	●				●	G		●		●				2019	[23]
	●					●					●				2020	[84]
Multiple cancers^e^	●					●	E		●		●				2017	[132]
Multiple cancers^f^	●	●				●		M	●		●				2018	[133, 134]
Ovarian cancer	●		●				G				●				2017	[135]
	●				●	●	G/scG	R	●	EM/Me	●			●	2019	[79, 136]
Pancreatic cancer	●	●		●	●	●	T/E	P	●						2015	[26]
	●	●		●	●	●	E	M/P	●	Me					2018	[81]
	●	●		●		●	E/G	R			●	●		●	2018	[82]
	●			●	●	●	G	R			●			●	2019	[137]
Prostate cancer		●				●	E	R	●	A	●				2014	[83]
Rectal cancer	●	●	●	●		●	E	P	●		●	●		●	2019	[138]
	●					●	E				●	●		●	2020	[84]

^a^*E*, whole-exome sequencing; *G*, whole-genome sequencing; *scG*, single-cell genome sequencing; *T*, targeted (cancer gene) sequencing

^b^*M*, microarray; *P*, qRT-PCR; *scR*, single-cell mRNA sequencing; *R*, mRNA sequencing

^c^*A*, array CGH; *EM*, electron microscopy; *Me*, DNA methylation analysis; *Pro*, proteomics

^d^Colorectal cancer, gastro-oesophageal cancer, liver cancer

^e^Bladder cancer, brain cancer, breast cancer, colorectal cancer, gastro-oesophageal cancer, kidney cancer, lung cancer, ovarian cancer, prostate cancer, small intestinal cancer, soft tissue cancer, and uterus cancer

^f^Breast cancer, digestive organ cancer, lung cancer, ovarian cancer, peritoneal cancer, and uterus cancer

## Molecular genetics meets cancer organoid technology

To better understand the molecular genetics of cancer, organoid cultures have been utilised for two complementary approaches. The first strategy is the mutational analysis of patient-derived cancer organoids via WGS, whole-exome sequencing (WES), or targeted sequencing for cancer gene mutations (Fig. 2). On the other hand, the second strategy aims at probing the consequences of specific mutations on tumourigenesis by introducing putative or validated cancer gene mutations into either normal organoids or cancer organoids using gene editing technology.

### Mutational analysis of patient-derived cancer organoids

As discussed above, cancer organoid biobanks were previously sequenced to confirm that cancer organoids robustly retain the genetics of the tumour epithelium on the bulk level (Table 1 and references therein). Mutational analysis of these biobanks revealed that the individual cancer organoid lines could be assigned to different molecular subtypes of cancer [72], which demonstrates that cancer organoids can represent inter-tumour heterogeneity in vitro. As normal organoid cultures remain largely genetically stable [36] and cancer organoids reflect the bulk genetic makeup of the cancer of origin [72], cancer organoid cultures allow for the investigation of clonal dynamics within cancer, a critical feature of intra-tumour heterogeneity and cancer responses to therapy. A recent study, for example, generated organoids from high-grade serous ovarian carcinoma tissue obtained from a single patient at different time points [79]. About 800 single-cell DNA sequencing profiles were generated from the primary tumour samples as well as corresponding tumour organoid lines from two different passages. For each single sequenced cell copy number variation (CNV), profiles were calculated and cluster analysis was performed. The analysis revealed that the primary tumour cells clustered into five different clusters [79]. All organoid cells were also assigned to one of the five clusters, suggesting that intra-tumour heterogeneity was maintained in vitro. Interestingly, one of the clusters contained diploid cells, and cells obtained from late passaged organoids were less abundant in this cluster. The authors suggested that these cells were likely normal cells that were outgrown by aneuploid tumour cells (present in the remaining four clusters) upon extended organoid culture [79]. Another study generated clonal organoid lines from several locations within CRC tissue as well as from adjacent normal colorectal epithelium from three different patients and analysed genome, epigenome, and transcriptome of each line [89]. WES analysis revealed a dramatically higher mutational load and a robust diversification of the mutational signatures in the CRC cells in comparison with the normal intestinal cells. Reconstruction of phylogenetic trees demonstrated that most mutations present in the cancer were absent from normal colorectal cells and were acquired in the late stages of clonal expansion [89]. Importantly, the mutational alterations likely formed the basis of the inter- and intra-tumour diversification, as epigenetic and transcriptional changes were aligned with the mutations present within each clonal CRC organoid line [89]. As this study highlights clonal (genetic) diversity within one cancer, it remains important for many applications of organoid technology in cancer research to consider and, if possible, prevent clonal drift in tumour organoids [90]. Here, clonal drift describes the process during which genetically diverse and polyclonal (cancer) organoid cultures reach a bottleneck with only one or a few dominant genetic clones surviving, as also seen in the homoeostatic small intestinal epithelium in vivo [91]. Different ways to minimise the risk of clonal drift in organoid cultures may be passaging the entire culture plate (instead of only a smaller fraction of it), returning to cryopreserved early passages of the cultures, or keeping the culture period as short as possible [38].

### Probing the role of (cancer) gene mutations in organoids

Epithelial organoid cultures are amendable to gene editing via different genetical tools, including CRISPR–Cas9 and RNAi [30, 40, 90, 92]. This versatility allows for the probing of specific gene functions in cancer formation or the modelling of cancer progression using organoids (Fig. 1). For instance, several studies have been published on the stepwise introduction of classical CRC driver mutations—*APC*^−/−^, *TP53*^−/−^, *SMAD4*^−/−^, and *KRAS*^G12D/+^—following the so-called Vogelgram cancer progression model [93]. Starting from patient-derived normal colon organoid cultures, the groups replicated key features of CRC progression, including independence of niche factors [88, 94], chromosome instability, aneuploidy, invasiveness [88], and ability to metastasise when the gene-edited quadruple mutated organoids were orthotopically transplanted into the caecum of NSG mice [95]. CRISPR–Cas9 gene editing of human colon organoids has further been applied to investigate the role of mutations in DNA mismatch repair genes such as *MLH1* [96] or *BRAF*^V600E^ mutations [97] in CRC. In another study, human liver ductal organoids were modified to harbour loss-of-function mutations for the tumour suppressor BAP1 [98]. To allow for selection of mutant organoid clones, the authors co-injected targeting plasmids for both the genes encoding BAP1 and TP53. As described above, Nutlin-3 could then be used to select for TP53 mutant organoids, which very likely were also BAP1 mutant due to the high efficiency of plasmid co-transfection observed in the system [98]. *TP53*^−/−^ *BAP1*^−/−^ double-mutant organoids showed abnormal morphology with loss of cell polarity, perturbation of the epithelial layer, and increased cell motility. Profiling of transcriptome and proteome provided further evidence for alterations in the expression of cytoskeletal and cell–cell junctional components, which are essential for the proper functioning of epithelia. In elegant rescue assays, the authors demonstrated that only catalytically active BAP1 localised to the nucleus can recover the homeostatic phenotype in the organoids [98]. When introducing other known liver cancer (cholangiocarcinoma) gene mutations in the organoids in combination with the *BAP1* mutation, the authors could show that BAP1 loss-of-function is required for tumourigenesis when mutant organoids are transplanted into mice [98]. The authors highlighted their study as an example to probe cancer gene function mechanistically in human tissue by combining organoid technology with gene editing tools such as CRISPR–Cas9 [98, 99]. An essential advantage of using organoid technology to probe the role of (cancer) gene mutations is the possibility to introduce the gene mutations of interest into a normal organoid line, which—in its unmodified form—serves as an isogenic control. This strategy helps to avoid artefacts introduced by the individual variability due to the genetic background of donors and should be carefully considered for such studies.

## Personalised anti-cancer therapy

Organoid technology is already exploited for personalised therapy of cystic fibrosis (CF) patients. CF is a monogenic disease caused by a wide spectrum of mutations in the cystic fibrosis transmembrane conductor regulator (CFTR) gene encoding a chloride ion transport channel [100]. An in vitro assay was developed using normal intestinal organoids generated from rectal biopsies of patients that allows for the prediction of patient response to CF drugs [101]. Following the proof-of-concept, several further studies validated the suitability of the drug testing platform utilising organoid cultures [102, 103]. Using different proxies to show inhibition of cancer cell growth or induction of cytotoxicity, limited drug screens have also been performed on cancer organoid biobanks (Fig. 2 and Table 1; [68]). However, robustness, reproducibility, and applicability of the assays to different cancer types still require further assessment before cancer organoid-based drug (or small molecule) screenings may be more broadly used for personalised medicine approaches [68, 87, 104, 105]. Another critical issue is whether patient-derived cancer organoids at all have the potential to predict patient response to anti-cancer therapy. Several studies aimed at resolving this concern comparing cancer organoid responses with chemo- or chemoradiotherapy with clinical outcomes. In one study, the authors generated fifty cancer organoid lines from metastatic tissues of different gastrointestinal cancers (i.e. CRC, gastro-oesophageal cancer, and cholangiocarcinoma) [87]. Following genotypical and phenotypical characterisation and validation of the cancer organoids as well as transcriptional profiling, the authors tested a variety of drugs directly in vitro on the organoids and in vivo using organoid-based murine xenograft models. Strikingly, when comparing their results with the clinical data, the authors found an 88% positive predictive value and 100% negative predictive value of organoid-based targeted therapy or chemotherapy [87]. Another study using organoids derived from metastatic CRC patients demonstrated almost similar predictive values for the drug irinotecan alone or in combination with 5-fluorouracil, while treatment with only oxaliplatin or oxaliplatin combined with 5-fluorouracil could not be validated [104]. A third study tested irradiation, 5-fluorouracil, and irinotecan on a set of rectal cancer-derived organoid lines and found a diagnostic accuracy of almost 85% using the organoids [84]. Lastly, a study described that cohorts of pancreatic cancer patients could be stratified based on transcriptional signatures and chemosensitivity profiles obtained from cancer organoid cultures of these patients [82]. Collectively, these efforts make a strong case that cancer organoids may have a significant predictive value for patient response to anti-cancer treatments. However, many variables, including the tumour microenvironment and drug metabolism or toxicity by peripheral organs, remain to be addressed.

## Immuno-oncology and the tumour microenvironment in a dish

The tumour microenvironment plays a critical role in tumour formation and progression (reviewed in [6]). Hence, the interaction of the tumour with its microenvironment is a heavily studied hallmark of cancer [106]. Bidirectional communication between tumour cells and cellular components of the tumour microenvironment such as fibroblasts, the vasculature, and immune cells plays a critical role in tumour promotion [106]. For instance, cancer cells may stimulate endothelial cells to induce angiogenesis or chronic inflammation mediated by tissue-infiltrating immune cells may provide survival factor or mitogens promoting tumour growth [106]. On the other hand, an active immune response may suppress tumour growth and, therefore, cancer cells may develop means to avoid immune destruction [106]. A better understanding of the influence of the tumour microenvironment on tumour growth dynamics is essential to guide anti-cancer therapy and minimise resistance against treatment [6]. However, the tumour microenvironment is composed of a heterogeneous pool of cells with a variety of features that may promote or prevent tumour growth, making it a highly complex biological system to be studied [6].

While cancer organoid cultures lack cellular components of the tumour microenvironment, they may serve as very good reductionist in vitro model systems to study the influence of tumour microenvironment on cancer growth. Recently, several immuno-oncological protocols have been developed using organoid technology (Fig. 2; reviewed in [43]). One such approach used cancer organoid co-cultures with peripheral blood mononuclear cells (PBMCs) to generate patient-specific tumour-reactive cytotoxic T cells [107, 108]. Since high degree of neoantigen presentation is critical to elicit a robust anti-tumour immune response by antigen-specific T cells [109, 110], the authors chose organoids generated from specific subtypes of CRC and non-small cell lung cancer with a high mutational burden. Through serial co-cultivation of cancer organoids and PBMCs in the presence of a T cell-stimulating growth factor cocktail, it was possible to select for and expand antigen-specific cytotoxic T cells in about half of the samples. Importantly, co-cultures of expanded T cells with organoid generated from adjacent healthy epithelial tissue resulted in undisturbed organoid expansion without significant levels of organoid cytotoxicity [107]. In another approach, tumour-infiltrating T cells and CRC organoids were separately expanded using their respective gold standard methods and then combined to assess T cell-mediated organoid killing [111]. Interestingly, the extent of cell death in the in vitro co-culture assay correlated well with the patient’s response to chemotherapy and immune checkpoint blockade [111]. Furthermore, the authors tested immune checkpoint blockade using PD1 antibodies in their co-culture model system and demonstrated that organoid killing by PD1^high^ T cells was improved upon antibody treatment. Two other groups described organoid-based immuno-oncology assays that utilised cytotoxic lymphocytes engineered to recognise defined antigens and kill organoids presenting such antigens. The first group generated chimeric antigen receptor-engineered natural killer cells recognising the antigens of choice [112], while the second group generated T cell receptor transgenic cytotoxic T cells [113]. In both cases, robust antigen-specific cytotoxicity was observed against cancer organoids presenting the antigen of choice [112, 113]. Co-cultures with matched normal epithelial organoids or cancer organoids presenting control antigens did not show significant levels of antigen-specific cytotoxicity. Apart from these reductionist approaches, other protocols to test lymphocyte-mediated tumour organoid killing have been established. One such approach utilises the air–liquid interface (ALI) cultures of patient-derived cancer organoids that preserved not only the tumour epithelium but also significant cellular components of the tumour microenvironment including fibroblasts, macrophages, and lymphocytes for about 1 month [114]. These ALI-based cultures could be established from various cancers such as CRC, lung cancer, head and neck cancer, and melanoma and also allowed for modelling of immune checkpoint blockade [114].

Apart from the immune infiltrate, fibroblasts may also critically contribute to tumour initiation and progression. A recent study in mice, for instance, described the presence of a rare population of *Ptgs2*-expressing fibroblasts that reside under the crypt epithelium and constitute the intestinal stem cell niche in mice [115]. The authors then showed that *Ptgs2*-expressing fibroblasts secreted prostaglandin E_2_ (PGE_2_), which promoted adenoma formation in the classical *Apc*^Min/+^ tumour mouse model in vivo [116]. As there is growing evidence for the tumour-promoting features of PGE_2_ [117, 118], but its cellular source remained elusive, the authors investigated further. To decipher the bidirectional signalling between stem cells and their mesenchymal niche, the authors then developed a co-culture method of mouse small intestinal organoids and wild-type primary mouse intestinal fibroblasts (Fig. 2). In the absence of fibroblasts, organoids started budding and displayed the typical crypt–villus architecture [11]. However, when co-cultured with fibroblasts, organoids formed cystic spheroids. This is usually only seen for a very short culture period or under high Wnt conditions with increased stemness and inhibited differentiation of the cultures [119]. By generating organoids lacking the main PGE_2_ receptor in the (murine and human) intestinal epithelium (prostaglandin E_2_ receptor 4, EP_4_; encoded by *Ptger4* in mice and *PTGER4* in humans), the authors then elegantly demonstrated that fibroblast-secreted PGE_2_ directly acts on the epithelial stem cells, as spheroids did not form when culturing *Ptger4*-depleted organoids [115], aphenotype that was readily reproduced by applying an EP_4_ inhibitor to the co-cultures [115]. Interestingly, PGE_2_ is a critical component of the growth factor cocktail for human intestinal organoid culture promoting stem cell proliferation and formation of the very characteristic cystic spheroids [16, 17]. In line with the results on murine intestinal organoids, the effect of PGE_2_ on human colon organoid cultures could also be blocked by PTGER4 inhibition [115], suggesting a conservation of the mechanism of action between mice and humans. Further along the line of tumour progression, cancer-associated fibroblasts (CAFs) play a major role in the tumour microenvironment, for example by providing mitogenic factors to the growing cancer as well as mediating resistance against anti-cancer therapy [6]. CAFs are typically studied following xenotransplantation of human cancer cells or organoids into mice; however, the tumour stroma generated in this setting is entirely composed of murine cells. In order to better understand the interaction between cancer cells and their mesenchymal niche, a few co-culture methods have been published over the last couple of years. One study, for example, described the development of a co-culture protocol of organoids and CAFs derived from pancreatic ductal adenocarcinoma (PDAC) to investigate stem cell niche factor dependency during tumour progression [81]. In their PDAC organoid biobank, the authors found that the organoids showed different levels of dependency on Wnt/R-spondin supplementation. Some organoids survived in the absence of both ligands, while others were fully dependent on exogenous Wnt ligands or both exogenous Wnt and R-spondin. The cellular source of Wnt ligands remained unknown. However, as PDACs are characterised by robust stromal cell infiltration, it was hypothesised that these may be the Wnt source promoting survival of Wnt-dependent PDAC subtypes in vivo. To test this, the authors went on to generate stroma-attached organoids by letting PDAC cells and CAFs (derived from the same patient) aggregate together [81]. In this co-culture system, Wnt-dependent PDAC organoids formed in the absence of Wnt supplementation [81], as Wnt3A was provided by the CAFs in short range to PDAC cells, in line with earlier studies describing a short-range Wnt gradient in intestinal organoids [120]. A similar effect was not observed when CAF-conditioned medium was provided or when Wnt-dependent PDAC organoids and CAFs were co-cultured without direct physical contact [81]. PDACs self-producing Wnt ligands have been shown to be more aggressive [81], which suggests that PDACs dependent on Wnt supplementation by CAFs may represent an initial stage of tumour initiation that is lost during tumour progression into an aggressive and metastatic cancer.

## Challenges and outlook

In order to use organoids for cancer therapy, several challenges remain to be overcome. ASC-derived organoid models have mostly been established from epithelial tissues, and, in line, cancer organoid cultures are in most cases only available for epithelial cancer such as different types of carcinomas with the exception of the recently described culture protocols for glioblastoma organoids [70] and rhabdoid tumour organoids [76]. Another critical issue is the efficiency at which cancer organoid cultures can be established [87], as well as the culture purity, as contamination with normal epithelial cells remains a problem, making organoid culture from some primary cancers such as prostate cancer very difficult [83]. Furthermore, to allow high-throughput assays, improved methods are required to decrease the time and costs of organoid generation as well as the input material needed to establish cultures. At the same time, other prerequisites for personalised (precision) medicine using cancer organoids are a better understanding of the clonal dynamics of cancers as well as the role of cellular components of the tumour microenvironment, which are too often still poorly understood. While some in vitro approaches have been developed to incorporate cells of the tumour microenvironment such as immune cells and fibroblasts into the cancer organoid culture [43, 107, 112, 114, 115], existing methods need further improvement and incorporation of more (non-epithelial) cell types. In addition, advancements to co-culture organoids with bacteria still need broader implementation and explorations. A major challenge remains the use of (non-human) animal products for organoid cultures such as murine-derived extracellular matrix (ECM) hydrogels (such as Matrigel, BME, Geltrex) or bovine-derived foetal calf serum or bovine serum in growth factor-conditioned media [68]. Bioengineering approaches such as the development of hydrogels using artificial matrices [121] or the use of alternatives to conditioned media may help to overcome some of these limitations in the future [122]. However, the need to test invasiveness or metastatic potential of cancer organoids using mouse xenograft models is still without robust alternatives [95]. Research into finding suitable replacements for such models should be fostered in the future. Lastly, ethical implications of cancer organoid biobanks preserving viable patient material require further considerations and may result in a stronger legislative regulation in the future [123].

Organoid technology has been developed just over 10 years ago. Its rapid implementation by numerous research groups worldwide led to many breakthroughs in the field of cell and developmental biology, but also in pre-clinical (cancer) research. The application of organoid technology in basic cancer research has provided many new experimental models and led to a variety of new discoveries. With the establishment of living biobanks of cancer organoids, new possibilities arise for the broader testing and development of anti-cancer drugs as well as the better stratification of cancer patient cohorts. With its versatility, robust ability to model in vivo situations, and fast-evolving set of applications, organoid technology is expected to keep making a significant impact on basic cancer research and clinical cancer therapy in the future.
